# Epithelial Predominant Wilms Tumor in an Adult Patient: Case Report and Literature Review

**DOI:** 10.15586/jkcvhl.v11i3.329

**Published:** 2024-08-12

**Authors:** Sofia Chapman, Benjamin Lichtbroun, Hiren Patel, Sai Krishnaraya Doppalapudi, Hatim Thaker, Colton Smith, Cristo Guardado Salazar, Scott Moerdler, Saum Ghodoussipour

**Affiliations:** 1 Section of Urologic Oncology, Rutgers Cancer Institute of New Jersey and Rutgers Robert Wood Johnson Medical School, New Brunswick, NJ;; 2 Department of Urology, Boston Children’s Hospital, Boston, MA;; 3 Department of Pathology, Immunology, and Laboratory Medicine, Rutgers Robert Wood Johnson Medical School, New Brunswick, NJ;; 4 Department of Pathology and Laboratory Medicine, Children’s Hospital of Los Angeles, Los Angeles, CA;; 5 Section of Pediatric Hematology and Oncology, Rutgers Cancer Institute of New Jersey and Rutgers Robert Wood Johnson Medical School, New Brunswick, NJ

**Keywords:** congenital anomalies, epithelial predominant, Jeune syndrome, renal tumor, Wilms tumor

## Abstract

Although rare in adults, Wilms tumor is the most common pediatric renal tumor. Treatment typically involves radical nephrectomy followed by adjuvant chemotherapy or radiation, although outcomes differ between children and adults which may be due to challenges in accurately diagnosing these patients. In this article, we present a case report of an adult patient with Jeune syndrome and multiple urologic abnormalities who underwent radical nephrectomy for a large renal mass and was subsequently diagnosed with an epithelial predominant Wilms tumor. Epithelial predominant Wilms tumor may have distinct origins from other Wilms tumor histological subtypes and may incur better outcomes. Herein, we discuss the literature surrounding this rare entity as well as the anticipated treatment course.

## Introduction

Wilms tumor (WT), also known as nephroblastoma, is the single most common tumor among children but only represents 5% of all adult renal malignancies. Historically, adult WT outcomes were much poorer, with some studies reporting that only 24% of adult patients were disease-free after 3 years as compared to 74% in children. However, when managed using pediatric protocols, a 2004 report by the National Wilms’ Tumor Study Group found the 5-year overall survival (OS) of adults improved to 83%, which was closer to the average pediatric cure rate of 90% using multimodal treatments (1). In 2019, the National Cancer Database compiled information on over 2500 cases in children, 91 cases in young adults (aged 16–35 years), and 35 cases in older adults (aged > 35 years). They found that the 5-year OS of children was 93.1% whereas in young adults and older adults, the OS was 79.1% and 78.9%, respectively. This study performed multivariate analysis with linear regression, comparing the rates of adjuvant therapy and lymph node dissection during surgical nephrectomy. They found that adults had decreased rates of chemotherapy (odds ratio or OR of 0.38, 95% CI 0.24–0.62), radiation therapy (OR of 0.62, 95% CI 0.4–0.95), and lymph node sampling (OR of 0.19, 95% CI 0.13–0.28) (2). The worse prognosis among adult patients could be explained by difficulties in diagnosing these patients, which then delays the proper treatment protocol (1, 3, 4).

Wilms tumor is often associated with certain congenital anomalies and chromosomal mutations, although differences exist between pediatric and adult WT. Pediatric WT is associated with *WT1* mutations, childhood overgrowth syndromes, tumor predisposition syndromes, and constitutional chromosomal abnormalities. Adult WT is associated with *WT1* and loss of heterozygosity (LOH) mutations as well as BRAF V600E mutations (2, 5, 6).

Our case showed a history of Jeune syndrome, an autosomal recessive disorder characterized by asphyxiating thoracic dystrophy because of osteochondrodysplasia. This thoracic insufficiency syndrome primarily involves skeletal abnormalities, including shortened limbs, shortened ribs, and a narrow thorax, which restricts respiration (7–9). Complications include renal, hepatic, pancreatic, gastrointestinal, and retinal abnormalities (8, 9). Renal complications previously seen with Jeune syndrome include renal hypoplasia, renal cystic dysplasia, pelviectasis, and renal failure. However, Jeune syndrome is not associated with renal tumors and specifically is not linked to either pediatric or adult WT previously (8, 10).

Treatment of adult WT relies on pediatric standards of care and varies based on staging and histology. Treatment plans generally involve radical nephrectomy followed by adjuvant chemotherapy or radiation if deemed necessary (1, 11, 12). Histologically, WT is composed of varying proportions of blastemal, stromal, and epithelial tissues. Patterns emerge based on the predominant tissue type and may indicate unique origins and improved outcomes among the epithelial predominant subtype (6, 11, 13). In this article, we present a case study of an adult patient who presented with a renal mass and history of Jeune syndrome with pathology at the time of surgery revealing an epithelial-predominant WT.

## Case Report

**Figure 1:**
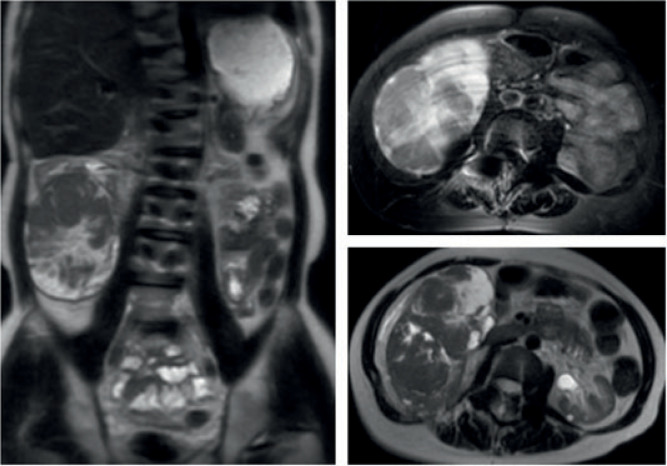
MRI imaging of abdomen without IV contrast: Multiplanar T1- and T2-weighted images of the abdomen were obtained without IV contrast. A large and complex mass is seen arising from the right kidney lower pole measuring 11.6 × 8.3 × 12.7 cm.

A 30-year-old male presented with a large right renal mass, discovered on renal ultrasound (US) performed due to a recent rise in creatinine levels from 2.0 mg/dL to 2.56 mg/dL with an estimated glomerular filtration rate (GFR) of 34. The renal ultrasound revealed a solid and heterogenous renal mass appearing 11.3 × 7.5 × 12.6 cm right side. He denied urinary complaints but noted vague abdominal discomfort. His past medical history included Jeune syndrome (asphyxiating thoracic dystrophy), chronic kidney disease (CKD), hypertension, hypercholesterolemia, retinitis pigmentosa, asthma, attention deficit hyperactivity disorder (ADHD), scoliosis, and obstruction of posterior urethral valves as well as a right uretero pelvic junction, both of which were repaired during childhood. His brother also had a past medical history of Jeune syndrome with renal failure, which required a kidney transplant. The family history was notable for appendiceal adenocarcinoma in his mother and prostate cancer in his father. The patient underwent a left renal biopsy as a toddler because of renal insufficiency, which revealed no gross abnormalities, and another renal biopsy at 29 years of age, revealing mesangial hyperplasia with focal glomerulosclerosis consistent with C1q nephropathy. Following the renal ultrasound, further work up with chest X-ray showed no evidence of metastases; however, magnetic resonance imaging (MRI) of the abdomen and pelvis without intravenous (IV) contrast revealed an 11.6- × 8.3- × 12.7-cm right lower pole mass with little normal kidney remaining (Figure 1).

**Figure 2:**
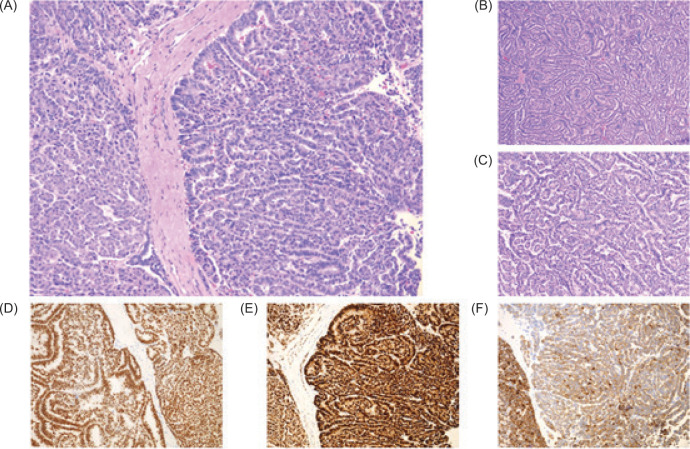
Kidney, right, radical nephrectomy (hematoxylin and eosin [H&E] staining). (A–C) Histologic sections showed variable degrees of epithelial differentiation ranging from more primitive rosette-like structures and cords to well-differentiated mature tubules lined by cuboidal to columnar cells with elongated nuclei; no significant blastemal or stromal component was identified. By immunohistochemistry, tumor cells were positive for (D) *WT1* and (E) *PAX8* with strong nuclear staining, and variable membranous and cytoplasmic expression of pankeratin I. Additional immunohistochemical staining for BRAF V600E mutation (not pictured) was negative. Overall, the histologic and immunohistochemical features were consistent with epithelial-predominant WT and favorable histology (absent anaplasia). Molecular testing revealed *TP53* gene mutation.

An open right radical nephrectomy was performed. Pathology revealed a 13.8-cm epithelial predominant WT with favorable histology, pT3aN0 (Figure 2). The tumor had invaded into the segmental branches of the renal vein. Margins were negative. One hilar lymph node was excised and was negative for malignancy, confirming the classification of a stage II WT. Brain MRI was performed and revealed an empty sella turcica, but no evidence of the disease. DNA sequencing revealed a nearly genome-wide copy-neutral loss of heterozygosity for chromosomes 1p, 11p15, 16q, and 17p13.1. Mismatch repair was intact and nuclear expressions of MLH1, MSH1, MSH6, PMS2, and programmed death-ligand 1 (PD-L1) were normal.

Typical adjuvant chemotherapy options for stage II WT, including vincristine, dactinomycin, and doxorubin, were discussed. Although these medications are not typically nephrotoxic, both patient and care team were concerned about organ damage, given the history of CKD with a post-operative GFR of 20 and his underlying genetic disorder.

Ultimately, the patient and family opted for close follow-up and surveillance imaging every 3 months for the first 3 years, then every 6 months for the following 2 years. It was decided to start by alternating an MRI of the abdomen/pelvis and chest computed tomography (CT) with an abdominal ultrasound and chest X-ray. After 2 years, the surveillance transitioned to abdominal ultrasound and chest X-rays alone. Positron emission tomography (PET) scans and contrast imaging were avoided due to the patient’s history of CKD.

The first set of screening tests included a chest X-ray and abdominal ultrasound, which was normal, except for the chest X-ray noting scoliosis and the abdominal ultrasound revealing cysts in the liver and left kidney and sludge in the gallbladder. The most recent follow-up appointment was 14 months post-surgery and revealed no evidence of the disease. Informed consent was obtained for the publication of this case report, and Institutional Review Board (IRB) approval was waived.

## Discussion

Wilms tumor is the most common renal tumor in children, with 8–10 cases per million children each year and is typically diagnosed around 3–4 years of age (1). Roughly 90% of all WT are present in children aged ,5 years and 95% are present in children aged <15 years (2). More than 500 children per year are diagnosed with WT in the United States (14). The exact incidence of WT in adults remains unknown but is estimated to be less than 0.2 per million per year with a median age of diagnosis in adults being 34 years (1). Owing to its infrequency in adults, less than 300 adult cases are published in the literature worldwide and no phase-3 studies or treatment standards are available (2).

Wilms tumor may present differently in adults compared to children. In children, WT is typically diagnosed by a palpable mass associated with painless hematuria. In comparison, adult patients tend to present with flank or abdominal pain, hematuria, or a palpable mass. Adult WT is typically larger and much more heterogenous (4). Metastasis is much more common among adults (29% of all cases), compared to 10% in children (15). The predominant sites of metastasis include the liver and lungs; nevertheless, metastasis can occur in the brain, bones, skin, bladder, large intestine, and contralateral kidney (15). The six criteria for diagnosing WT in adults were established in 1980 and include the following: the patient must be aged >15 years; pictorial confirmation of histology is present; the tumor is a primary renal neoplasm with primitive blastematous spindle or round cell component; abortive or embryonal tubular or glomeruloid structures are formed; and that no area of the tumor is diagnostic of hypernephroma (4).

Pediatric WT is associated with numerous congenital diseases and chromosomal abnormalities. The British National Registry of childhood tumors found that 9% of pediatric patients with WT also demonstrated at least one congenital anomaly, some of which include but are not limited to horseshoe kidney, cardiac septal defects, and cervical rib abnormalities (5). A retrospective case series conducted by Dumoucel et al. found that 17.6% of children with WT had either a clinically identified malformation or a predisposition syndrome (16).

Conditions found to have a significantly increased risk in children include *WT1* deletions and missense mutations, such as WAGR (acronym for Wilms tumor, aniridia, genitourinary malformations and a range of mental disabilities) syndrome (50% risk of developing WT) and Denys–Drash syndrome (up to 75% risk of developing WT), familial WT (2% of all WTs have a family history), Perlman syndrome (55% of those who survive infancy develop WT), mosaic variegated aneuploidy (roughly 85% risk), and Fanconi anemia (20–60% risk) (5, 17, 18). Conditions associated with a slightly increased risk include Beckwith–Wiedemann syndrome (4% risk), Simpson–Golabi–Behmel syndrome (3% risk), Bloom syndrome (3% risk), *WT1* splice mutations (Frasier syndrome), Li-Fraumeni syndrome, hereditary hyperparathyroidism–jaw tumor syndrome, Mulibrey nanism, Trisomy 13 and 18, and 2q37 deletions (5, 18).

Certain biomarkers are associated with an increased risk of developing pediatric WT and poor prognosis. Notably, LOH on 11p15 has an increased risk of recurrence with a hazard ratio (HR) of 5.0 (2.8–7.2). An LOH on 1p and 16q was also linked to increased risk of recurrence (HR 2.93 and 1.95, respectively). LOH at chromosome 1p was found to occur in 10% of pediatric WT patients, and 20% of pediatric WT have an LOH at 16q. Combined LOH 1p/16q may prompt clinicians to pursue more aggressive radiation therapy or chemotherapy to mitigate the risk of recurrence (14). A recent study conducted by Hol et al. discovered that 8.9% of pediatric WT patients had mutations in genes that predisposed adult-onset cancer, such as *PMS2, CHEK2*, and *MUTYH* genes (19). Several studies have discovered that 14–29% of WT was associated with upregulation of PD-L1, and a recent retrospective observational study conducted by Zhang et al. found that 35% of metastatic WT was associated with an upregulation of PD-L1 (20, 21). Interestingly, in our case, nuclear expression of PD-L1 was normal; however, an LOH for chromosome 17p13.1 as well as for 1p, 11p15, and 16q was determined.

While WT in adults has been linked to many genetic alterations, especially mutations in *WT1* (present in roughly 10% of adult cases) and an LOH at chromosome 11p, adult WT has not yet been linked to developmental disorders or above-mentioned genitourinary malformations (2, 6). Roughly, 15% of WT in children are syndromic; however, only one adult with WT was identified to have a *WT1* germline mutation and one adult WT was revealed in a patient with cryptorchidism and hypospadias. Notably, our patient did show a history of genitourinary anomalies, including posterior urethral valves and an ureteropelvic junction obstruction as well as a history of Jeune syndrome.

Microscopically, WT appears similar in both children and adults with varying amounts of blastema, stroma, and epithelial cells. However, adult WT is more often blastemal-predominant, presents as more heterogenous, and more often has regions of necrosis and hemorrhage (2, 4). Each portion of the triphasic pattern has distinguishing features. Blastema is typically the least differentiated tissue and tends to be the most malignant (13). It is characterized by small cells with round nuclei, small nucleoli, and scarce cytoplasm (2). The epithelial cells vary greatly from very little differentiation to structures that resemble tubules or glomeruli and may include mucinous tissues or squamous epithelial islands. Lastly, the stromal portion may be made up of two sub-components with varying packing patterns, including dense undifferentiated mesenchymal cells and loose cellular myxoid tissue. This histologic heterogeneity complicates diagnosis, as each tumor may contain drastically different morphology (13, 22). Compared to pediatric WT, adult WT is not associated with nephrogenic rests (NR), clusters of embryonal cells, which are regarded as precursor lesions of WT in pediatric populations. Interestingly, bilateral WT is heavily associated with nephrogenic rests and make up roughly 6% of pediatric WT; however, less than 0.5% of adult WTs are bilateral (6, 23).

Epithelial-predominant WTs are especially challenging to distinguish from other neoplasms, including metanephric adenoma (MA). Epithelial-predominant WT may demonstrate small, highly differentiated, and tightly packed tubules which resemble MA (22). Interestingly, several cases are diagnosed in which an epithelial-predominant WT contained distinct regions histologically identical to well-differentiated metanephric adenoma. BRAF V600E mutations are observed in more than 90% of all metanephric adenomas, which is more common in adults and is more often observed in adult WT as well (6, 24). In a recent study conducted by Argani et al., 5/14 or 35% of their adult WT cohort demonstrated BRAF V600E mutations, although the investigators warned that two of the five patients were sent to them specifically because of their interest in this WT subset (6). All five patients with this mutation were categorized as epithelial-predominant with metanephric adenoma-like regions. The cited study also had five additional patients of epithelial-predominant tissue without BRAF V600E mutations or metanephric adenoma-like regions (6). This intends that BRAF V600E mutations and regions of metanephric adenoma are more common in epithelial-predominant WT but not necessarily present in all epithelial-predominant WTs (6). Another recent study conducted by Pan et al. observed that whole transcriptome sequencing revealed more similarities between epithelial-predominant WT with metanephric adenoma regions and typical metanephric adenoma, compared to the typical monophasic epithelial WT (24). This evidence may suggest a common BRAF-mutated pathway which leads to epithelial WT from a metanephric adenoma origin (24). Although this was not observed in our patient, BRAF mutations hold clinical significance, as BRAF inhibitors, including vemurafenib and dabrafenib, may be implemented in treatment (25). The cited study also identified three patients of monophasic epithelial-predominant WT negative for BRAF V600E mutations. Two of these three patients were adults and the third was 13 years old, which is much older than the typical age range of pediatric WT. The somatic copy number alteration (SCNA) patterns in these three cases were distinct from both typical WT and typical metanephric adenoma. These findings suggest that adult epithelial-predominant WT, much similar to the above-described our patient, may be distinct from both pediatric WT and the BRAF V600E-mutated epithelial-dominant WT with regions of metanephric adenoma (24).

In the United States, WT in children is treated with immediate nephrectomy and risk-dependent adjuvant therapy. However, because WT is so rare among adults, specifically no standard treatment protocols for adults are present. Current practice is to apply the pediatric guidelines to adult patients (3). The Children’s Oncology Group (COG), which is generally followed in the United States, recommends radical nephrectomy followed by risk-adapted adjuvant therapy 1–2 weeks post-nephrectomy to decrease recurrence (3, 26). In general, the poorer prognosis among adults may be attributed to the relative rarity of WT in adults, which induces clinicians to overlook it as a diagnostic possibility. Renal cell carcinoma (RCC) is a far more common adult renal tumor and is difficult to distinguish from WT using radiological findings alone, leading to a high incidence of initial misdiagnosis (3). Depending on the radiographic appearance, size, and growth kinetics of the mass, RCC may be managed with active surveillance and applying the same treatment protocol to WT, a more rapidly growing tumor, allows the tumor extra time to develop (27). Adult WT is thus more often diagnosed at an advanced stage, with 50% described as stage III–V at the time of diagnosis (1). While many adults with renal masses are not established with a medical oncologist prior to surgery, a diagnosis of WT on final pathology may prompt the need to initiate care for the first time, delaying the adjuvant therapy which would have ideally been administered within 1–2 weeks post-surgery (3, 27). This delay in adjuvant therapy may incur poorer outcomes (1–4). A retrospective cohort study determined that on average adults with WT were given adjuvant therapy 59 days post-surgery. In the cited study, the authors also discovered that the patients given treatment within 30 days of diagnosis had a 5-year event-free survival (EFS) of 60% whereas the patients with a delay of >30 days had a 5-year EFS of 14.3% (P = 0.03) (1).

Despite the need for adjuvant therapy in higher-risk presentations, it comes at a cost and must be evaluated using a risk-dependent approach to minimize adverse effects in patients who may not require it (3). Historically, 24% of all WT survivors are impacted by negative effects of treatment, including cardiac or pulmonary toxicities, infertility, or secondary malignancies (11). Generally, pediatric patients with stage I tumors are given vincristine and dactinomycin (1). However, nephrectomy alone has also been effective in children with very low-risk WT, which requires a stage I favorable histology tumor weighing less than 550 g, as determined in a patient aged <24 months (12). Interestingly, 25% of very low-risk WT are epithelial-predominant. A study conducted by Parsons et al. in 2020 analyzed 177 pediatric patients with WT and determined no difference in EFS among stage I epithelial-predominant favorable histology WT prescribed vincristine and dactinomycin treatment postoperatively, compared to observation alone (11). The authors discovered a 4-year EFS in patients given adjuvant therapy versus observation alone of 96.1% (95% CI 90.8–100%) and 98.2% (95% CI 92.8–100%, P = 0.55), respectively (11). The 4-year OS of both groups was 100%. Only six events were reported in 177 cases of stage 1 epithelial-predominant favorable histology WT. Three of these events were tumor development in the opposite kidney. The remaining three events were metastatic disease within regional lymph nodes, liver, and/or lung. Notably, all three of these metastases occurred in patients who had received chemotherapy as opposed to observation alone. However, two of these three patients did not have lymph nodes sampled during nephrectomy, indicating that metastasis could have always been present and improper staging had occurred (11). Several prior studies revealed similar results, with one study reporting a 5-year EFS of 90.2% and an OS of 98.4% in epithelial-predominant WT, compared to 84.0% and 92.5%, respectively, in other histological subtypes (28–30). In our case, adjuvant therapy was typically indicated. However, the recent literature highlighting positive outcomes among epithelial-predominant favorable histology WT, along with consideration of our patient’s comorbidities, including CKD, helped guide our decision to treat with nephrectomy alone, followed by close monitoring for recurrence. Adjuvant therapy was avoided to mitigate potential harm in a histological subtype that may not require it to accomplish a positive outcome.

## Conclusion

Wilms tumor is a rare tumor among adults, making its diagnosis difficult and delays treatment, which may lead to poorer outcomes, compared to children. Owing to its low incidence among adults, a finite number of cases are documented globally with limited literature available. Understanding the association with congenital anomalies would allow clinicians to have a higher index of suspicion for WT in these patients, which may allow for earlier diagnosis and treatment. Although adult WT appears to differ in some ways from pediatric WT, using the same treatment protocols as that for children, adult WT could have better outcomes than observed in the past. Importantly, stage I epithelial-predominant favorable histology WT has encountered very promising EFS and OS even without the need for adjuvant therapy, compared to other histological subtypes. This grants clinicians options in the effort to reduce the negative impact of adjuvant therapy. Epithelial-predominant WT’s association with metanephric adenoma and BRAF V600E mutation may provide clinicians alternative treatment options.
